# Porphyrin-Based Supramolecular Flags in the Thermal Gradients’ Wind: What Breaks the Symmetry, How and Why

**DOI:** 10.3390/nano11071673

**Published:** 2021-06-25

**Authors:** Angelo Nicosia, Fabiana Vento, Giovanni Marletta, Grazia M. L. Messina, Cristina Satriano, Valentina Villari, Norberto Micali, Maria Teresa De Martino, Maaike J. G. Schotman, Placido Giuseppe Mineo

**Affiliations:** 1 Department of Chemical Sciences and INSTM UdR of Catania, University of Catania, Viale A. Doria 6, I-95125 Catania, Italy; fabiana.vento@phd.unict.it (F.V.); gmarletta@unict.it (G.M.); grmessi@unict.it (G.M.L.M.); cristina.satriano@unict.it (C.S.); 2 LAMSUN-CSGI Unit of the Interuniversity Consortium for the Development of Large Interphases Systems (CSGI), Università di Catania, Viale A. Doria, 6, I-95125 Catania, Italy; 3 Consorzio Interuniversitario di Ricerca in Chimica dei Metalli nei Sistemi Biologici (CIRCMSB), Università Degli Studi di Bari Aldo Moro, I-70121 Bari, Italy; 4 Institute for Chemical and Physical Processes, National Research Council (IPCF-CNR), Viale F. Stagno d’Alcontres 37, I-98158 Messina, Italy; villari@ipcf.cnr.it (V.V.); micali@ipcf.cnr.it (N.M.); 5 Department of Chemistry & Chemical Engineering, Eindhoven University of Technology, P.O. Box 513, 5600 MB Eindhoven, The Netherlands; m.t.d.martino@tue.nl; 6 Institute for Complex Molecular Systems, Laboratory of Chemical Biology, Eindhoven University of Technology, 5612 AZ Eindhoven, The Netherlands; m.j.g.schotman@tue.nl; 7 Institute of Polymers, Composites and Biomaterials, National Research Council (IPCB-CNR), Via P. Gaifami 18, I-95126 Catania, Italy

**Keywords:** porphyrin aggregates, spontaneous symmetry breaking, asymmetric thermal gradients, PEGylated porphyrin derivative, thermally induced circular dichroism in stagnant solution

## Abstract

The Spontaneous Symmetry Breaking (SSB) phenomenon is a natural event in which a system changes its symmetric state, apparently reasonless, in an asymmetrical one. Nevertheless, this occurrence could be hiding unknown inductive forces. An intriguing investigation pathway uses supramolecular aggregates of suitable achiral porphyrins, useful to mimic the natural light-harvesting systems (as chlorophyll). Using as SSB probe supramolecular aggregates of 5,10,15,20-tetrakis[*p*(ω-methoxypolyethyleneoxy)phenyl]porphyrin (StarP), a non-ionic achiral PEGylated porphyrin, we explore here its interaction with weak asymmetric thermal gradients fields. The cross-correlation of the experimental data (circular dichroism, confocal microscopy, atomic force microscopy, and cryo-transmission electron microscopy) revealed that the used building blocks aggregate spontaneously, organizing in flag-like structures whose thermally-induced circular dichroism depends on their features. Finally, thermal gradient-induced enantioselectivity of the supramolecular flag-like aggregates has been shown and linked to their size-dependence mesoscopic deformation, which could be visualized as waving flags in the wind.

## 1. Introduction

On every scale of the Universe, the Spontaneous Symmetry Breaking (SSB) phenomenon happens. It is an event through which nature chooses the handedness of one species over the opposite, apparently without any reason [1,2,3,4,5]. But also in laboratory experiments, when achiral molecules are subjected to self-organization or supramolecular aggregation, the phenomenon has been noted [6,7,8]. The porphyrin derivatives are among the most used types of SSB probes [7,9] because of their well-known characteristics, such as high molar absorptivity and water solubility (induced by peripheral charged groups and/or hydrophilic polymer branches), their ability to self-aggregate by tuning the solution chemical-physical properties (such as pH or concentration [10,11,12]), and their sensitivity towards external physical stimuli (such as stirring) [13]. Moreover, the porphyrin aggregates have established themselves as an essential model because these systems result in a powerful light-harvesting system that mimics the chlorophyll antenna system [14]. The light absorption and the solar/chemical energy conversion capability of the chlorophylls aggregate systems found their best expression in many natural systems (plants, bacteriochlorophyll, and in all photosynthetic organisms). Notably, the aspects regarding the light-matter interaction are fascinating because they could provide a deeper understanding of many natural phenomena that could have determined the enantioselectivity in the ancestral biosynthesis of life’s building blocks (amino acids, carbohydrates, etc.) [15,16,17,18].

Many papers demonstrating the role of the aggregate (H or J) type and/or the geometry arrangement (tubular, toroidal, fractal) in the interaction with the light have been published [19,20,21,22,23]. An important issue is the necessity to work in aqueous media (useful to mimic the natural systems). An approach to solve the problem of porphyrin insolubility in aqueous media is binding ionic groups (sulphate, carboxylate, pyridinio, etc.) in its peripheral positions. Nevertheless, the nature of the interactions between the ionic dyes to forming aggregates species is different from the natural chlorophyll systems: the first interact mainly using anion-cation ionic groups (managed by varying pH and ionic strength); the second interact through cooperative polar, hydrophobic, and π-π interactions. Due to the strong electrostatic force, the ionic porphyrins may not represent a suitable way to mimic the interactions in natural systems. 

In this framework, to overcome the issue due to charged moieties, our workgroup already synthesized a particular class of non-ionic water-soluble porphyrin derivatives [24], with PEG chains in the meso-positions of a tetrakis-*p*-hydroxyphenyl-porphyrin. These porphyrin derivatives in an aqueous solution spontaneously produce H- and J-aggregates [25,26,27,28].

**Figure 1:**
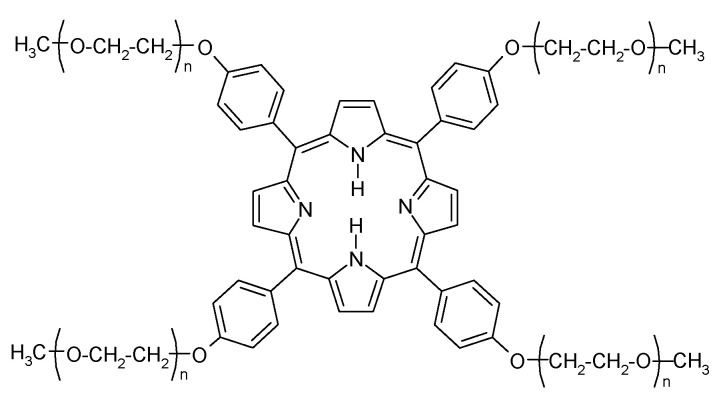
Structure of StarP.

Focusing on a symmetrical star polymer, the 5,10,15,20-tetrakis[*p*(ω-methoxy-polyethyleneoxy)phenyl]porphyrin (StarP, Figure 1), we have found that its aggregates show interesting characteristics useful to study the Symmetry Breaking (SB) phenomenon. Indeed, it has been demonstrated that, differently from porphyrinic charged ones, applying on its stagnant solution a weak asymmetric thermophoretic force [29,30], it is possible to manage the enantiomeric enrichments (observable using circular dichroism technique). Also, in pyrene 2D aggregates obtained by different preparation methods [31], we were able to find a correlation between aggregate structural properties and chiral response to either hydrodynamic or thermophoretic asymmetric perturbation. These results led us to further investigations focusing only on the size and morphology of the aggregates, excluding the role of methodology of sample preparation. 

Considering the lack of literature -reported data about size-dependent studies of supramolecular porphyrin aggregates referring to neutral water-soluble porphyrin derivatives, we focused on the structure-effect correlation between the supramolecular aggregates size and the SB phenomenon. Besides, the study might aim to help the comprehension of some aspects related to the spontaneity of the symmetry breaking events occurring in nature. In this work, using the StarP aggregates as an SB probe, families of aggregates different in size are individuated and investigated in depth. Their spectroscopic properties and their chiral response under temperature gradient fields (acting as external asymmetric perturbations) are revealed by UV-Vis and Circular Dichroism spectroscopies. The morphology and size of the aggregated supramolecular system have been investigated through AFM, TEM, and Dynamic Light Scattering. The behavior of the isolated supramolecular aggregates families has been characterized by means of MALDI-TOF Mass spectrometry and Dynamic Light Scattering. The obtained data have been correlated with UV-Vis and Circular Dichroism results.

It is shown that only the meso- and micro-sized aggregates, for which the structure can take a morphology of ribbon-shaped sheets (flag-like), display enantio-response to weak asymmetric thermal gradients perturbations. Finally, the possible chiral deformation of the supramolecular structure has been determined through cryo-TEM experiments and observed through confocal laser microscopy. To our knowledge, this is the first work ever reporting a mesoscopic waving behavior involved in a thermal-induced SB phenomenon.

## 2. Materials and Methods

All the solvents used in this work were purchased from Sigma-Aldrich (Merck Group, Milan, Italy). 

MALDI-TOF mass spectra were recorded in linear mode, using a Voyager DE (PerSeptive Biosystem, Perkin Elmer, Waltham, MA, USA) using a previously reported procedure [32,33] (25 kV, time delay = 2600 ns, potential gradient = 454 V/mm, wire voltage = 25 V) and equipped with a nitrogen laser (337 nm, 3 ns) and an AD converter working at 500 MHz. Trans-3-indoleacrylic acid (IAA) was used as a matrix. The calibration was performed as reported in a previous article [34]. Average molecular weights were determined using a Grams/386 software (Version 3.04, Galactic Industries Corp, Salem, NH, USA), following a previously reported method [35]. The *m*/*z* values reported in the text refer to molecular ions, considering the most abundant isotope of each element in the molecule. 

UV-Visible spectra were acquired using tetrahydrofuran (THF) or water as solvents at 25 °C, in quartz cells (optical path length of 1 cm for water and 0.5 cm for THF), using a Shimadzu Model 1601 spectrophotometer (Shimadzu Corporation, Kyoto, Japan). 

The circular dichroism spectra were acquired using a J-815 spectropolarimeter (Jasco Corporation, Tokyo, Japan) having a 150 W Xenon lamp as a light source. The ellipticity calibration (θ ∝ e_L−_ε_R_) of the instrument was obtained using a 0.06% (w/v) aqueous solution of ammonium d-10-camphorsulfonate and with a 0.08 percent (w/v) aqueous solution of tris (ethylenediamine)-Co complex (2(-)_D_-[Coen_3_]Cl_3_·NaCl·6H_2_O). The measurements were performed using water as a solvent, employing heating and cooling thermal ramps (range 5 °C–30 °C, 1 °C/min), in quartz cuvettes having 1 cm optical path (4 windows Hellma cell) with low birefringence [30]. Spectra were corrected considering the contribution of the cuvette and the solvent. The temperature control of the cuvette holder was performed using a Jasco PTC-423S/15 Peltier-type temperature control system [30]. 

Dynamic Light Scattering (QELS) measurements were acquired using a miniDAWN Treos multi-angle light scattering detector (Wyatt Technology, Santa Barbara, CA, USA), equipped with a Wyatt QELS-DLS module. The data analysis was performed with the ASTRA software (version 6.0.1.10, Wyatt Technology, Santa Barbara, CA, USA).

Atomic Force Microscopy (AFM) measurements were carried out with a commercial Nanoscope IIIA-Multimode AFM (Digital Instruments, Santa Barbara, CA, USA). Topographic (height) and phase images were recorded in tapping mode under ambient conditions, maintaining the force at the lowest possible value by continuously adjusting the set point during imaging. A total of 0.5–2 Ω·cm^−1^ Phosphorous (*n*)-doped silicon tips mounted on cantilevers with a nominal force constant of 40 N·m^−1^ and a resonant frequency of 300 kHz were used for these measurements. Images were collected at a scan rate of 1.5 Hz and at a scan angle of 0°. Measurements were made at least three times in each case in the middle area of the sample and the image analysis was carried out using DI software (version 5.31r1 (Digital Instruments, Santa Barbara, CA, USA). The images were flattened to remove background slopes. The thin films analyzed were deposited by drop-casting of 5 µL StarP water solution (5µM) upon a Polymethylhydrosiloxane (PMHS)/silicon substrate.

TEM images were recorded by a FEI Tecnai 20 (type Sphera) (FEI Company, Eindhoven, The Netherlands) at 200 kV. A total of 5 μL of the 5µM StarP water solution was dropped onto a carbon-coated copper grid (200 mesh, Electron Microscopy Sciences, Hatfield, PA, USA). Samples were left to dry at RT overnight. Two different magnifications have been used for the transmission electron microscopy, resulting in two scale bars for the images: namely 2 µm and 500 nm.

For cryo-TEM measurements, Lacey carbon film grids (LC200-CU) were used (Electron Microscopy Sciences, Hatfield, PA, USA). Before sample addition, grids were surface plasma-treated at 5 mA for 40 s using a Cressington 208 carbon coater. Using an automated vitrification robot (Vitrobot Mark III) (Thermo Fisher Scientific, Waltham, MA, USA), 3 µL sample was applied to the grids and excess sample was removed by blotting using filter paper for 3 s at –3 mm. The thin film formed was vitrified by plunging the grid into liquid ethane just above its freezing point. On a FEI-Titan TEM equipped with a field emission gun operating at 300 kV (FEI Company, Eindhoven, The Netherlands) the samples were examined. Post-GIF (Gatan imaging filter) 2k × 2k Gatan CCD camera (Gatan, Pleasanton, CA, USA) was used for the recording of the images. Micrographs were taken at low dose conditions, using a defocus setting of 20 μm at 11.5 k magnification or defocus setting of 50 μm (or 40 μm) at 6.5 k magnification. In particular, for the images shown in Figure 13: a,b: defocus −20 μm, 11.5 k magnification; c: defocus −50 μm, 6.5 k magnification; d: defocus −40 μm, 6.5 k magnification.

Laser scanning confocal microscopy (LSM) analyses were carried out on an Olympus FV1000 microscope (Olympus, Tokyo, Japan), equipped with diode and gas (Ar multiline and HeNe) lasers and oil immersion objective (60× O PLAPO). A spectral filtering system was used and the detector gain was fixed at a constant value. Images were collected in sequential mode at random locations throughout the area of each sample. For LSM-spectroscopy, the spectral filtering was set on beam split mode and scan sequences (xy-lambda mode, 5 nm interval) were acquired. Afterwards, the emission spectra were reconstructed from selected regions of interest (ROIs) on the recorded images.

Synthesis of 5,10,15,20-Tetrakis[p(ω-Methoxypolyethyleneoxy)Phenyl] Porphyrin (StarP)

StarP was synthetized employing ω-methoxy-polyethyleneoxy chloride (Mn = 350 Da, Polydispersity Index (PDI) = 1.01) and 5,10,15,20-tetrakis-(*p*-hydroxyphenyl)porphyrin as described elsewhere [36].

## 3. Results

The 5,10,15,20-tetrakis[*p*(ω-methoxy-polyethyleneoxy)phenyl]porphyrin macromolecular system (StarP, Figure 1) is a water-soluble PEGylated porphyrin derivative having bonded PEG chains with Mn = 350 Da. This particular average polymerization degree (in averaging Xn = 9) balances the hydrophobic/hydrophilic ratio of the porphyrin-PEG system, allowing the self-assembly process. Instead, longer PEG chains, due to the higher hydrophilic nature and shielding the porphyrin planes, favor the solubilization of the system [28]. Thanks to the PEGylation, this system show a particular behavior in aqueous solution than common ionic porphyrins (i.e., TPPS): it has a non-ionic structure; it is soluble in a large class of solvent (water, toluene, acetone, chloroform, THF, etc.); it spontaneously aggregates in water solution, but not in organic solvents. 

All the experiments were performed at a concentration of about 6 μM because this concentration ensures the negligibility of the amount of monomer species in solution than that of H- and J- aggregates. Besides, higher porphyrin derivative concentration would induce supramolecular inter-aggregation phenomena, reducing the amount of isolated supramolecular aggregates, which is an unwanted issue in microscopies experiments. 

**Figure 2:**
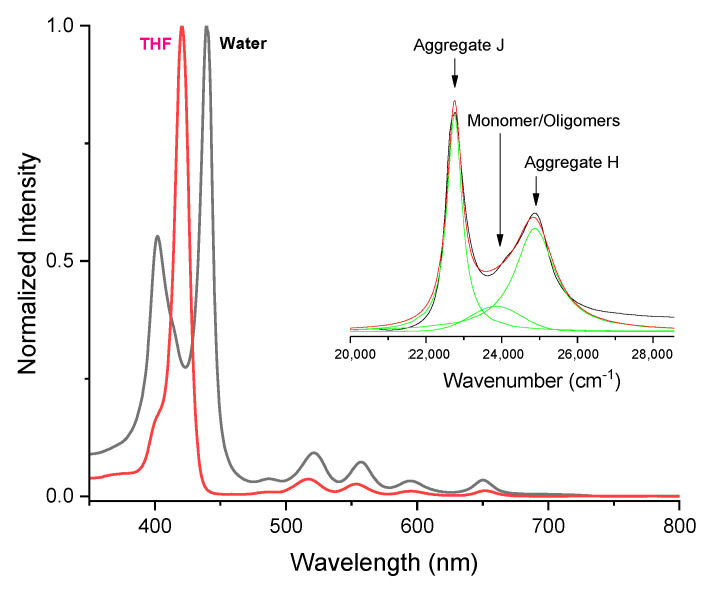
Normalized UV-Vis spectra of the water (black line) and THF (red line) solutions of StarP (~6 µM in both solvents). In the inset, deconvoluted curves (green lines) and fitted curve (red line) of the UV-Vis spectrum of the StarP aqueous solution (black line).

The UV-Vis absorption spectrum of StarP in THF solution (Figure 2, red line) shows a Soret band at 421 nm and four Q-bands at 517, 553.5, 595, 651.5 nm, respectively. According to the already reported Gouterman’s four orbitals model [37], the Q-band number is interpreted as the low-lying (π, π*) excited states of porphyrins in terms of electronic transitions between the two topmost filled molecular orbitals (HOMO’s), a_2u_ (π) and a_1u_ (π), to two degenerate lowest empty molecular orbitals (LUMO’s), e_g_ (π*).

Instead, the UV–vis spectrum of the water solution of StarP (Figure 2, black line), exhibits the splitting of the Soret band into two and the appearance of two new bands showing blue- and red-shifts, compared to the Soret band observed in THF. Several phenomena could give rise to the red-shifted signal, such as the protonation of the porphyrin core [38,39,40,41,42], the bathochromic effect of the solvent [43,44], the flattening of the porphyrinic phenolic moieties [45], or the aggregation phenomena [46]. Nevertheless, the first three phenomena should be excluded because: (a) the experiments were performed in water as it is (pH 6.5); (b) the solvent effect on the Soret band is, generally, small [43,44]; (c) twisting and flattening of the aromatic substituents are expected to be hindered due to the presence of the large polyether groups that prevent the free rotation of the phenolic groups.

Also, in the light of these considerations, the observed shifts were attributed to the exciton coupling between the porphyrin cores, with the formation of H- (face-to-face) and J-type (side-to-side) aggregates [47,48,49] with the Soret band splitting [50] in two new bands at 402 and 440 nm, corresponding to the H- and J-aggregates bands, respectively. Moreover, the typical four Q-bands at 521, 557, 595, 650 nm of the free base porphyrins are also evident in the spectrum. It is noticeable that in aqueous solutions, StarP instantly aggregates spontaneously (no environmental conditions to manage to induce the aggregation), on the contrary to the ionic porphyrin derivatives. As previously demonstrated [28,51,52], the aggregation phenomenon is driven by the hydrophilic/hydrophobic ratio of the PEGylate porphyrin system, due to π-π stacking interactions of porphyrin cores and the dipolar PEG-chains contribute.

The deconvolution of the UV-Vis spectrum of the StarP water solution was performed (see inset in Figure 2) to investigate the aggregation phenomenon in water. The obtained curves show the simultaneous presence of both H- and J- aggregates species in solution together with a small amount of monomeric and/or oligomeric species.

**Figure 3:**
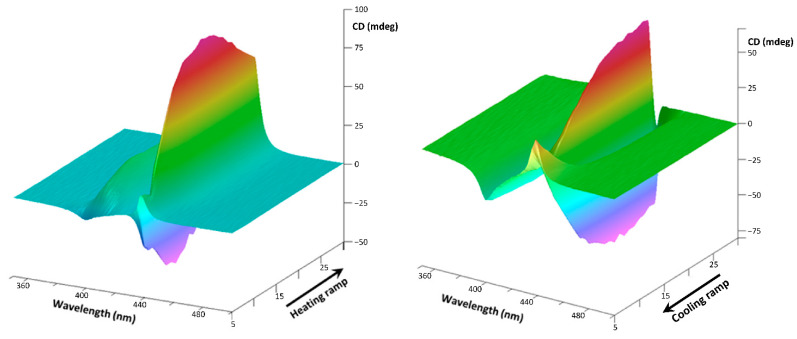
CD spectra collected during heating (from 5 °C to 30 °C, **left**) and cooling (from 30 °C to 5 °C, **right**) ramps of the StarP water stagnant solution (6 µM).

When immersed in weak asymmetric thermophoretic fields (in water stagnant solution), the supramolecular aggregates of StarP show a thermally induced CD signal (t-ICD) due to the Symmetry Breaking (SB) phenomenon occurring [29,30]. The asymmetry of the thermophoretic fields is generated through the Peltier based cuvette holder used. Thanks to its peculiar geometry [29,30], the six facets of the cuvette exhibit small temperature differences. Thus, when heating and successive cooling thermal ramp at 1 °C/min is applied (Figure 3, left and right, respectively) to the StarP water stagnant solution, the J-aggregates CD signal exhibit a reversible splitting Cotton effect [30], identifying an SB phenomenon induced by the weak asymmetric thermophoretic forces [29]. 

**Figure 4:**
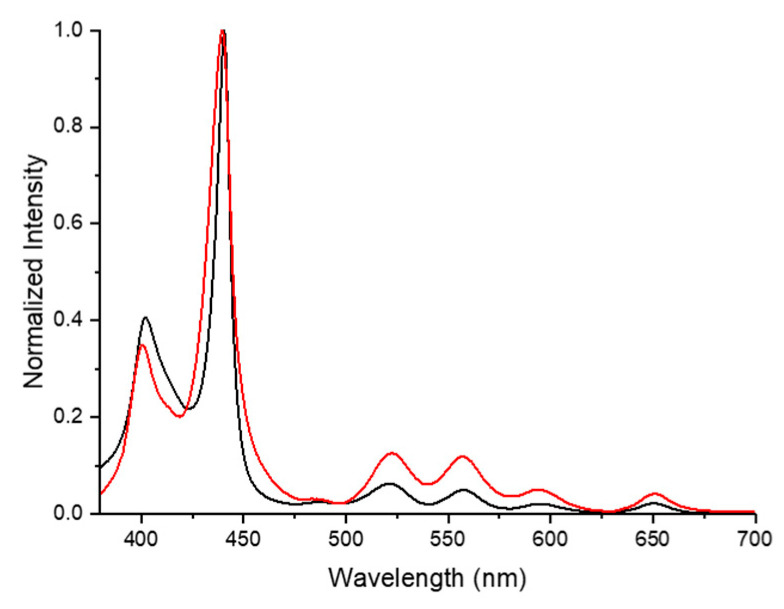
Normalized UV-Vis absorption spectra of the StarP in aqueous solution (black line) and thin film deposited on PHMS surface (red line).

Information on the shape and morphology of the aggregates is obtained from AFM and TEM experiments. The structural stability of the H- and J-aggregates formed in solution has been checked when these aggregates are allowed to “land” onto solid surfaces. Accordingly, UV-Vis spectra were obtained for a solution of StarP (5 µM) drop-casted on PHMS surface (Figure 4, red line).

The UV-Vis profile, similar to the one of the StarP aqueous solution (Figure 4, black line), confirmed that the drying procedure preserves the H- and J-aggregates structure. 

**Figure 5:**
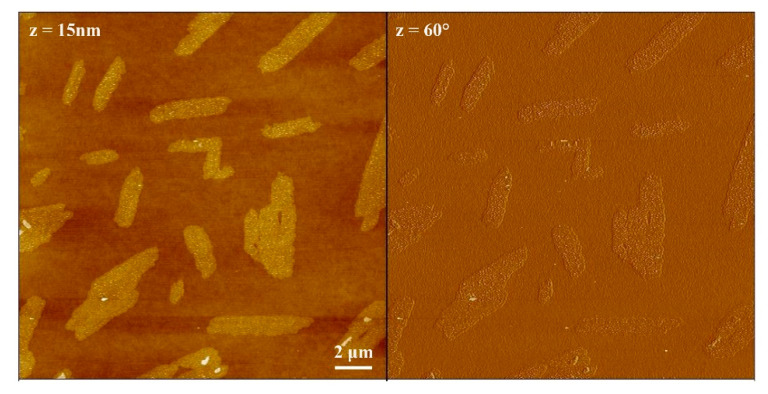
AFM topographies of StarP deposited on PHMS/Si: height image (**left**) and phase image (**right**).

Figure 5 reports the AFM images showing the nanoscale morphology of these aggregates deposited on hydrophilic atomically smooth PHMS surfaces, obtained by spin coating and drying process, followed by a UV-ozone treatment, which, while maintaining the original smoothness at the nanometer scale, allowed to optimize the wettability of the surfaces [53]. In fact, after the UV-ozone treatment, the roughness of the polymer surface is 0.28 ± 0.01 nm and it is completely hydrophilic, having a water contact angle under 5°. The drop-casting of the StarP solution, and drying, allow the deposition of a set of flat rectangularly-shaped aggregates, similar to “flakes”, thick, in average, about 2.4 ± 0.3 nm, with a width three orders of magnitude larger 1.2 ± 0.2 μm and a length ranging between 1.4 ± 0.1 μm and 6.3 ± 0.3 μm and another set of larger and longer structures, having similar thickness and length, but larger width, ranging from about 1.9 μm to 2.7 μm. The aggregates of this second set are probably due to the merging of the smaller “flakes” in a flat-on arrangement. 

However, by considering the single porphyrin molecule dimension, consisting of a central portion about 1.8 nm long, with the four branched PEG chains folded in random coil conformation, we suggest that the thickness of the flakes, registered above, is due to a misaligned and tilted side-to-side arrangement of StarP molecules, which assemble in J-aggregates. 

Phase images, providing a measure of the tip-surface friction, generally allow obtaining a “rough” chemical mapping of the surface, based on materials differences registered as bright and dark regions. Notably, in the phase image (Figure 5, right) there is no color difference, suggesting that the sampled material (i.e., the StarP molecules) on the whole surface is the same.

**Figure 6:**
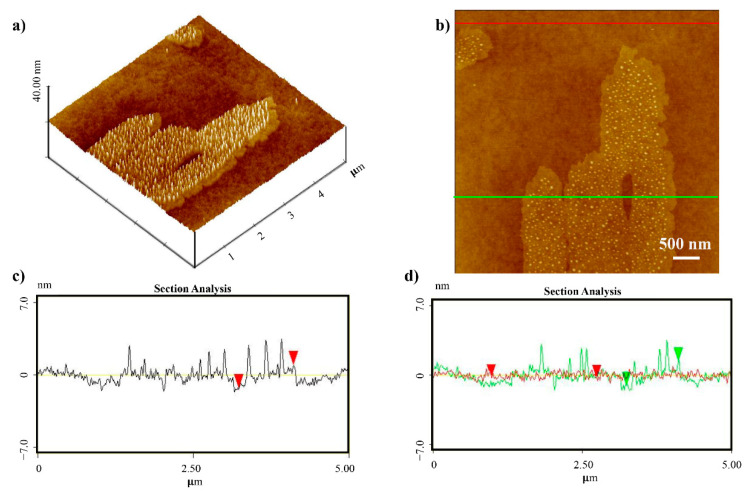
AFM 3D topographies (**a**) of the thin film of StarP deposited on PHMS/Si and relative profile (**b**–**d**).

To better investigate this aspect and the layer deposited at the interface, a magnification 5 × 5 μm as well as the 3D image and the related section analysis, have been performed and shown in Figure 6. It is possible to see the detailed structure of the “flakes” consisting of a porphyrin uniform layer and very small circle aggregates on top of it. The roughness of the surface free from StarP “flakes” was measured, resulting in about 0.31 ± 0.2 nm, i.e., a little bit higher than the treated polymer ones, suggesting the formation of a first non-assembled StarP molecular layer, in turn promoting the formation of the self-assembled “flakes”. The section analysis (Figure 6c,d) highlights both the flake thickness and the surface roughness in agreement with this finding. 

TEM images have been acquired on a dried solution of StarP to validate the AFM data by using two different magnifications.

**Figure 7:**
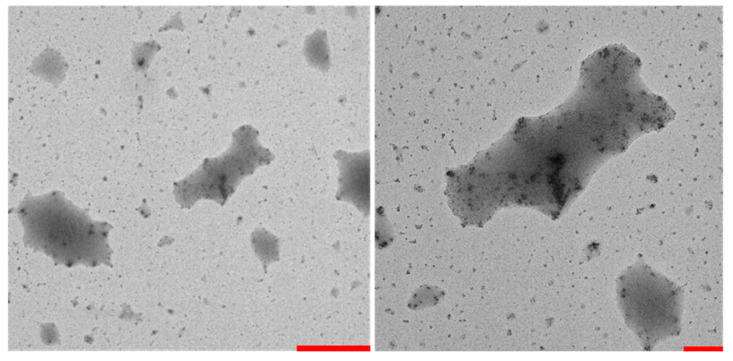
TEM images of the StarP thin film: (**left**), scale bar corresponds to 2 μm; (**right**), scale bar corresponds to 500 nm.

Experimental TEM images confirm the elongated rectangular-like shape of the aggregates (Figure 7), which are very similar, in size and shape, to the one observed with AFM. In particular, the samples analyzed essentially show the arrangement of large and thin sheets in the micrometric size (light grey); on the other hand, also nano-aggregates are present, isolated and/or overlapped with these sheets.

Despite both AFM and TEM analyses confirming the hypothesized aggregate structure, it is not clear if these supramolecular sheets are preorganized in solution or formed during the de-wetting phenomenon. To dispel any doubts and confirm the size distribution of the supramolecular aggregates of StarP in water solution, Dynamic Light Scattering (DLS) measurements were performed. 

Although the average size of the supramolecular aggregates of StarP was determined previously [28] (showing a fractal-like structure with R_g_~0.6 µm, R_H_~0.37 µm), the obtained average values could hide some important information on the number and size of the supramolecular aggregates present in aqueous solution. Thus, a new set of DLS experiments was performed to investigate in-depth the size distribution of the supramolecular StarP aggregates.

**Figure 8:**
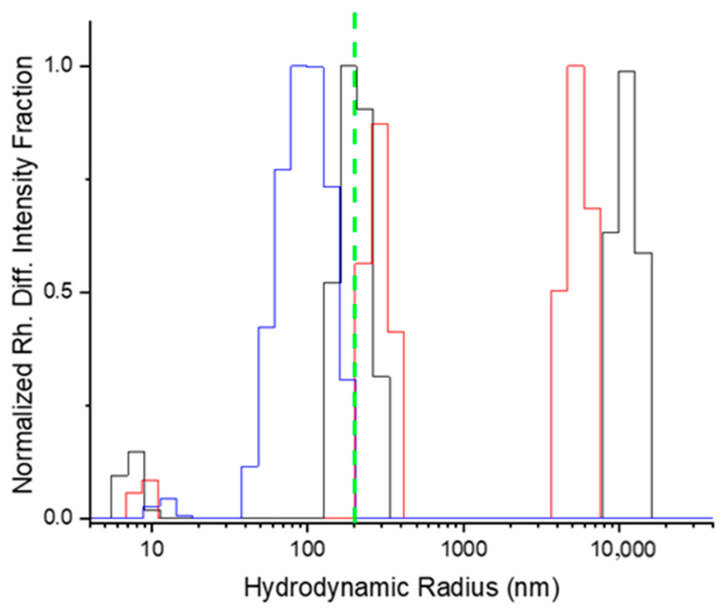
Normalized Differential intensity fraction as a function of the hydrodynamic radius of the aggregate species in water solution: parent (red line), StarP-R (black line), and StarP-F (blue line). The threshold of the filter (radius 225 nm) is also reported (green dashed line).

The DLS data show that the supramolecular aggregates of the StarP have a multi-modal dimensional distribution (Figure 8, red line). There are three families of aggregate structures having hydrodynamic radius (R_H_) of about 10, 300, and 6000 nm, respectively. 

The smallest objects (R_H_ = 10 nm) could be aggregates formed by a few building blocks, but their low scattering contribution does not allow for a reliable determination of the average size. The presence of aggregates ranging from micro- to nano-size is in accordance with AFM and TEM data, suggesting that the drying processes are not influencing the aggregates’ multi-modal size distribution.

Therefore, the multi-modal (micro- and nano- metric) size distribution of the supramolecular species in solution must be considered to define whether the size of the supramolecular aggregates species is responsible for the interaction with weak asymmetric thermal gradient fields. Thus, the isolation of the different families of aggregates was performed by filtration. 

A filter with 0.45 μm diameter pores was used to separate in size the aggregates, producing two samples: the filtered solution (StarP-F, cut-off size < 0.45 μm) and the residue (cut-off size > 0.45 μm); this last was recovered through counter-current washing of the filter with water (StarP-R). 

The separation of the aggregated species was confirmed by DLS analyses. The DLS measurement of the StarP-R solution (Figure 8, black line) shows a multi-modal distribution. Instead, the StarP-F solution (Figure 8, blue line) exhibits essentially a nanometric species having an average radius of ~200 nm, thus confirming a rough separation in size. 

**Figure 9:**
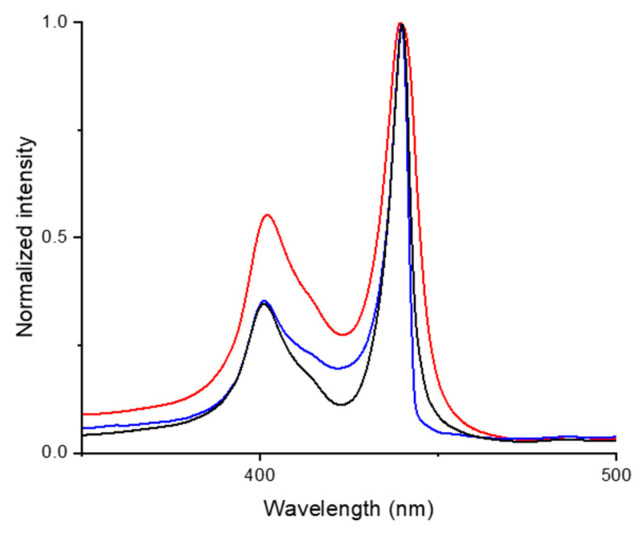
Normalized UV-Vis spectra of StarP water solution (red line), and of the StarP-F (blue line) and StarP-R (black line) solutions.

Moreover, through UV-Vis analysis (Figure 9), the persistence of the J-aggregates species despite the filtration process was verified. Indeed, the absorption profile of the parent sample and those of StarP-F and StarP-R solutions are, essentially, similar in terms of absorption wavelength and therefore of H- and J- aggregate structure. It is worth noting that StarP-R and StarP-F solutions do not exhibit significant differences in signal intensity and relative amount of H- and J-aggregates. 

**Figure 10:**
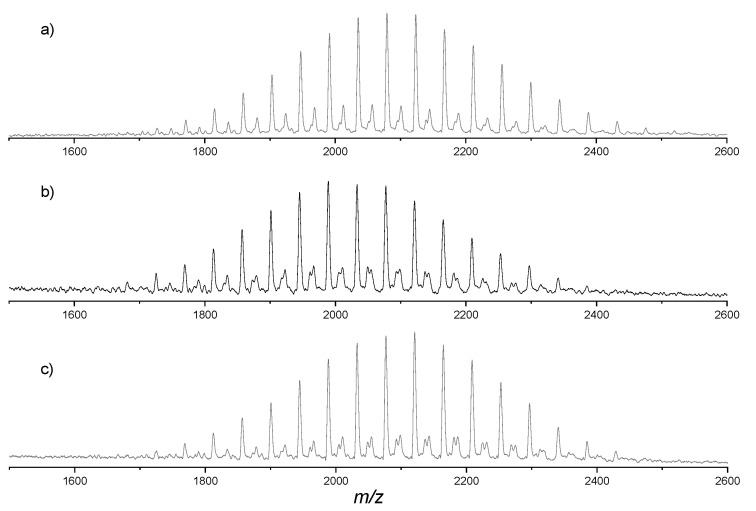
MALDI-TOF mass spectra of the samples: parent (**a**), StarP-R (**b**), and StarP-F (**c**).

MALDI-TOF mass spectrometry analysis was conducted on the parent, StarP-F, and StarP-R solutions, respectively, to define the structure of the isolated building blocks that compose the different supramolecular aggregates (Figure 10). 

The positive MALDI-TOF mass spectrum of the StarP aqueous solution before the filtration procedure (parent) is reported in Figure 10a. The spectrum consists of a cluster of peaks in the mass range 1650–2500 Da, centered at about *m*/*z* 2080 Da ($\bar{M_{w}}$ =2080 Da and $\bar{M_{n}}$ =2069). As expected, the peaks are separated by 44 uma as a consequence of the increasing amount of oxyethylene units in the porphyrin PEG branches. In particular, the peaks result at *m*/*z* value of 1681 + *n*× 44 with *n* = 21–40 (*n* indicating the total number of oxyethylene units in the four branches of StarP). These peaks correspond to the molecular species cationized with Na^+^, while peaks due to protonated and potassiated species appear with very low intensity at *m*/*z* values 1659 + *n*× 44 and *m*/*z* 1697 + *n*× 44, respectively.

The MALDI-TOF mass spectra of StarP-R (Figure 10b) and StarP-F (Figure 10c) show a similar peaks distribution to that of the parent solution, but with molecular mass distribution shifted at lower mass for the StarP-R sample (mass range 1650–2380 Da, centered at about *m*/*z* 2020 Da, $\bar{M_{w}}$ = 2015 Da and $\bar{M_{n}}$ = 2003 Da) and at higher mass for the StarP-F one (mass range 1725–2430 Da, centered at about *m*/*z* 2100 Da, $\bar{M_{w}}$ = 2076 Da and $\bar{M_{n}}$ = 2065 Da). These data suggest that macromolecular systems having (on average) shorter PEG branches form bigger aggregates (>0.45 μm). It is not clear, at the moment, if such a slight decrease of the average polymer chain length of the branches can, alone, affect the aggregation phenomenon acting on the hydrophilicity/hydrophobicity ratio of the supramolecular building blocks. Nevertheless, similar influences on the aggregation phenomenon have been previously identified [28].

**Figure 11:**
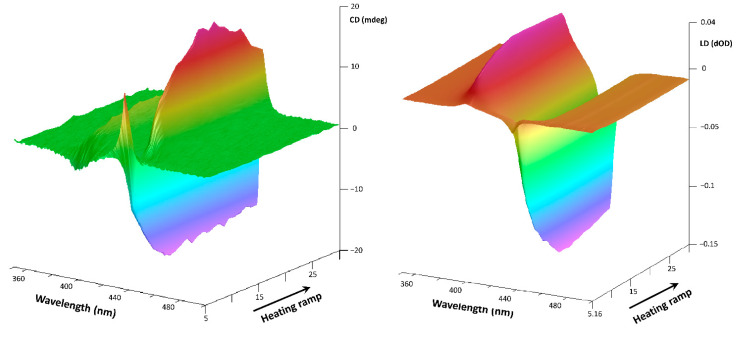
3D CD spectra of the water solution of StarP-R, acquired in thermal heating ramp (**left**) and the relative 3D LD spectra (**right**), in the temperature interval 5–30 °C, relatively to the aggregates having size >0.45 μm.

**Figure 12:**
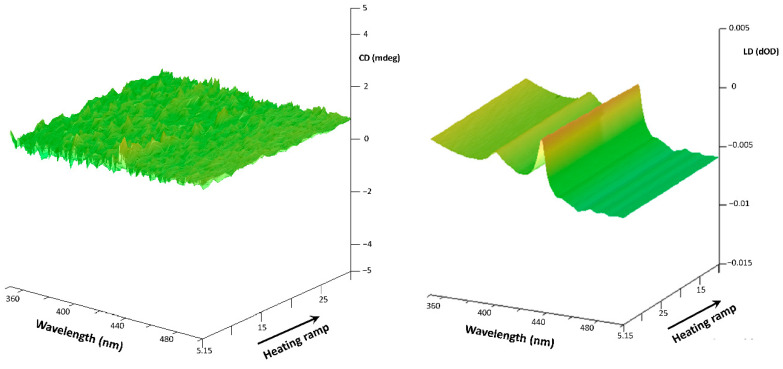
3D CD spectra of the water solution of StarP-F, acquired in heating thermal ramp (**left**) and the relative 3D LD spectra (**right**), in the temperature interval 5–30 °C, relatively to the aggregates having size <0.45 μm.

To understand which of the isolated aggregates species (nano- or micro-metric) are involved in the SB phenomenon, the CD experiments in a thermal ramp were conducted instantly after the filtration procedures. The experimental results are shown in Figure 11 and Figure 12. 

It is evident as the microscopic aggregates of StarP-R interact with the asymmetrical thermal fields, determining bi-signed t-ICD signals: the thermophoretic flows induce the enantiomeric enrichment revealed as a splitting Cotton effect (Figure 11, left). Moreover, an LD contribute is also detected (Figure 11, right), indicating the spatial alignment of the aggregates under perturbation. However, it has to be underlined that such an alignment does not affect the CD signal significantly. Indeed, the LD signal retains its sign upon an inversion of the thermal ramp, whereas the CD signal changes its sign, as shown in Figure 3. Besides, the negligibility of the cross-talk between the LD and the CD calculated previously for the parent solution [29] suggests that no artifacts are present in the CD spectra of the present work.

On the other hand, although UV-Vis measurements verified a similar molar concentration of the StarP-F solution than that of the StarP-R, the StarP-F solution does not show t-ICD signals, but only a weak LD signal [54] (Figure 12). Consequently, the chirality of the nanoscopic aggregates (<0.45 μm) is not influenced by the asymmetric thermophoretic fields.

Cryo-TEM experiments were performed to shed light on the shape of the StarP supramolecular aggregates in water solution and the possible pathway for their asymmetric deformation.

**Figure 13:**
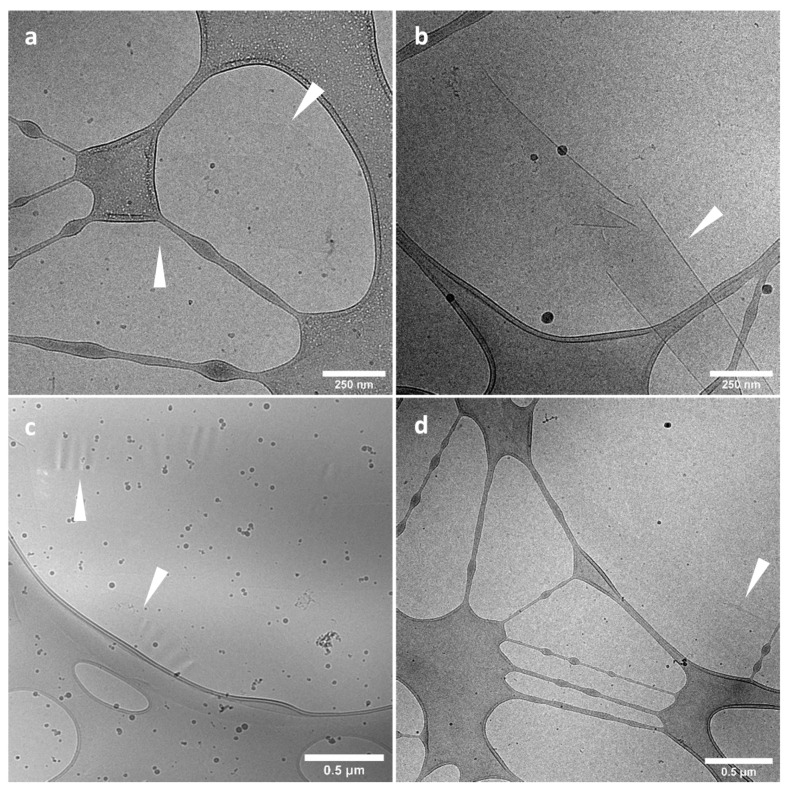
Cryo-TEM images (**a**–**d**) of the StarP aggregates in water solution.

Cryo-TEM images of the vitrified specimens show long ribbon-like structures (Figure 13a), with widths ranging from nano- to micro-meters. A number of these structures are noticeably stacked on top of each other (Figure 13b). Although these structures have a large surface area, appearing to be straight and flat, it is noteworthy to say that some of these sheets exhibited surprising flexibility, with curved structures (Figure 13c), as well as partially folded edges (Figure 13d).

The formation of the above-described curved structures may be due to the occurred stimuli during the sample preparation and deposition on the grid, with consequent asymmetrical deformation of the aggregates.

Therefore, thanks to their shape, these supramolecular ribbons could act as flags during the interaction with the thermophoretic fields: their large surface could intercept several asymmetric flows, different in strength, which modify their supramolecular shape in a chiral way. 

Because these microscopic aggregates change their supramolecular chirality under the asymmetric solicitations and lose it when the thermal flows cease [29], seeming to exhibit elasticity [55] when thermal ramps are applied in solution, the macroscopic structure could stretch and/or twist under a weak external stimulus reversibly. 

Given the reported results, it emerges that aggregate size is certainly important in determining the CD response to asymmetric perturbations, but also the shape and morphology play a significant role. In particular, the existence of large thin aggregates possessing a certain degree of flexibility (mainly due to weak supramolecular interactions among the building blocks, like hydrophobic, π-π, and hydrogen bond) is a strong requisite. 

We hypothesize that the flat supramolecular ribbons, in stagnant solution and fine-thermostated conditions, have statistical local deformations that are reflected in the absence of circular dichroism signal. Instead, when thermal ramps are applied in solution, the macroscopic structure could have, dynamically and reversibly, local asymmetric deformations. The handedness excesses of these deformed areas consequently result in a transient partial offset orientation excess of porphyrin-based building blocks [56], which is reflected in the recorded circular dichroism signal.

Contrarily, the nanoscopic structures are just directing by the directions of the flow (as the Brownian motions) [30].

Taking into account previous investigation on modified-pyrene achiral aggregates [31], the data here reported support the hypothesis that the CD response to asymmetric perturbations is correlated only to the supramolecular features of the aggregates, regardless of the chemical nature of the molecule involved in the aggregates.

**Figure 14:**
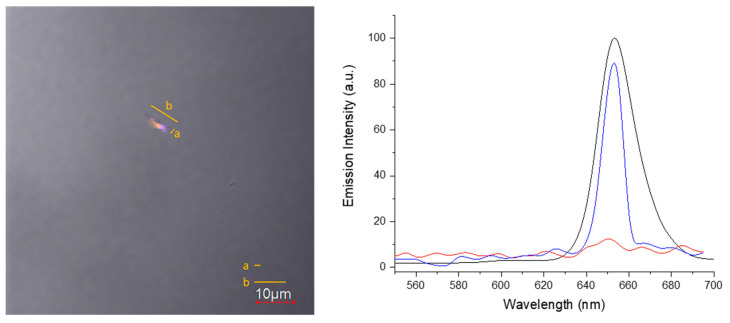
**(left**), merged optical bright-field image and confocal micrograph (emission at 650 nm) of a StarP aqueous solution. (**right**), fluorescence emission spectra of the confocal microscopy region of interest with (blue line) and without (red line) StarP aggregate, at λ_exc_ 405 and 543 nm, and the emission profile of the StarP aqueous solution (black line).

Confocal microscopy was used to observe the behavior of the supramolecular aggregate in water solution at room temperature (Figure 14). 

The overlapped optical and fluorescence images (Figure 14, left) of the StarP water solution showed an isolated aggregate with a rectangular shape similar to the ones observed above by AFM, TEM Cryo-TEM images, with an average length of ~8 μm and an average width of ~1 μm. Moreover, as reported in Figure 14-right, the fluorescence emission at 650 nm (λ_exc_ 405 and 543 nm) confirms the porphyrinic nature of the observed microscopic aggregated structure.

Finally, the time-lapse video (see Video S1), acquired with both optical and fluorescence channels on the solution of StarP, shows how the detected microscopic aggregate is locally deforming in the recorded time. Also, nanoscopic aggregates floating under Brownian motion are detectable in the fluorescence channel of acquisition.

## 4. Conclusions

This study was focused on the relation between the supramolecular aggregates features and the SB phenomenon. Thermally-induced Circular Dichroism was revealed on StarP water solution subjected to weak asymmetry thermal gradient fields. The aggregates’ morphology was investigated in dry conditions through AFM and TEM experiments, revealing that micro-sized aggregates organize themselves into ribbon-like shapes with different sizes. DLS studies evidenced a multi-modal size distribution of the supramolecular aggregates, whose families can be separated through filtration procedures. Subsequent CD experiments suggest a dependence between the supramolecular aggregates’ size and the thermally induced SB phenomenon. 

Finally, Cryo-TEM experiments confirmed that the micro-sized aggregates, in water solution, organize themselves into ribbon-like shapes and show local deformations. The occurrence of the supramolecular aggregates motions has also been revealed by confocal laser microscopy in water solution. 

Based on the evaluated experimental data, an interpretation to explain how the supramolecular micro-aggregates interact with the weak asymmetric thermal gradients in the solution was elaborated. 

Because of the large surface of the microscopic aggregates, they intercept different thermal gradients and, as a consequence, the inhomogeneous thermal flows induce the asymmetric deformation of the supramolecular structure. To make the idea, aggregates intercepting the thermal flows behave similarly to flags in the wind, as shown by Cryo-TEM experiments (Figure 13c) and confocal microscopy (Figure 14 and Video S1). This phenomenon will result in an enantiomeric enrichment of the population of the species, revealed as CD splitting Cotton effect.

Instead, the nano-sized aggregates do not intercept different thermal flows’ intensities, determining a partial orientation with the direction of the thermal flows, generating only LD effect.

The results exposed in this work represent a first approach towards the comprehension of the structure-effect correlation of supramolecular aggregates and the SB phenomenon, useful to shedding light on the spontaneity of the symmetry breaking events occurring in natural systems. As here demonstrated, the cooperative interactions of the supramolecular systems define a supramolecular minimum cut-off size to which the chiral effects induced by weak external stimuli undergo. Thus, the “spontaneity” in symmetry breaking events might be just illusory for low-sized supramolecular aggregates.

## Data Availability

Data are not available due to further project advancement.

## Supplementary Materials

The following are available online at https://www.mdpi.com/article/10.3390/nano11071673/s1, Video S1: time-lapse video of StarP solution, acquired with both optical and fluorescence channels, showing the local deformation of a microscopic aggregate during the recorded time.
